# Local Maps of the Polarization and Depolarization in Organic Ferroelectric Field-Effect Transistors

**DOI:** 10.1038/srep22116

**Published:** 2016-02-24

**Authors:** Ronggang Cai, Alain M. Jonas

**Affiliations:** 1 Bio & Soft Matter, Institute of Condensed Matter and Nanosciences, Université catholique de Louvain, Croix du Sud 1/L7.04.02, B1348 Louvain-la-Neuve, Belgium

## Abstract

We study the local ferroelectric polarization and depolarization of poly(vinylidene fluoride-*co*-trifluoroethylene) (P(VDF-TrFE)) in p-type ferroelectric field-effect transistors (FeFETs). Piezoresponse force microscopy (PFM) is used to obtain local maps of the polarization on model metal-semiconductor-ferroelectric stacks, and on FeFETs stripped from their top-gate electrode; transfer curves are measured on complete FeFETs. The influence of the semiconductor layer thickness and of the polarity and amplitude of the poling voltage are investigated. In accumulation, the stable “on” state consists of a uniform upward-polarized ferroelectric layer, with compensation holes accumulating at the ferroelectric/semiconducting interface. In depletion, the stable “off” state consists of a depolarized region in the center of the transistor channel, surrounded by partially downward-polarized regions over the source and drain electrodes and neighboring regions. The partial depolarization of these regions is due to the incomplete screening of polarization charges by the charges of the remote electrodes. Therefore, thinner semiconducting layers provide higher downward polarizations, which result in a more depleted transistor channel and a higher charge injection barrier between the electrodes and the semiconductor, leading to lower threshold voltages and higher on/off current values at zero gate bias. Clues for optimization of the devices are finally provided.

Since the first organic memory ferroelectric field-effect transistor (FeFET) was demonstrated by Naber *et al.* in 2005^1^, organic non-volatile memory FeFETs have become a promising and active topic, evidenced by increasing numbers of scientific publications and applications^1^,^2^,^3^,^4^,^5^,^6^,^7^,^8^,^9^,^10^,^11^. The structure of a typical FeFET is given in Fig. 1c. Here, we report for the first time on direct microscopic measurements of the spatial distribution of polarization in the ferroelectric layer of a real organic FeFET, and on the effect of the semiconducting thickness on the stability of the polarized states.

In organic FeFETs, poly(vinylidene fluoride-*co*-trifluoroethylene) (P(VDF-TrFE)) is the most often used ferroelectric material, due to its high remnant polarization, short switching time, and low processing temperature (typically 120–140 °C)^12^,^13^. As the memory functionality of a FeFET uses different polarization states of the ferroelectrics to store information, the polarization and depolarization behavior of P(VDF-TrFE) has been studied in FeFETs under different conditions, *e.g.*, varying device configurations, changing poling conditions, and using different organic or inorganic semiconductors^1^,^2^,^4^,^6^,^8^,^14^,^15^,^16^. A common conclusion from these previously-published studies is that, when the semiconductor is in the accumulation regime, the accumulation charges compensate the polarization charges of the ferroelectric layer in contact with the semiconductor (Fig. 1c), thereby decreasing the electrostatic energy. After removing the poling voltage, the polarization remains stabilized by these mobile compensating accumulation charges, leading to high source-drain current values. Starting from this polarization state, depolarization only occurs when the gate bias exceeds the coercive voltage of P(VDF-TrFE).

In contrast, when the semiconductor is poled in the depleted state, different ferroelectric polarization behaviors have been reported and the details of the mechanism remain elusive. For instance, Naber *et al.* reported that the polarization switches between a stable polarized state and a depolarized state in metal-ferroelectric-semiconductor-metal (MFS) devices with organic poly(3-hexylthiophene) (80 nm) as semiconductor. These two states correspond to the accumulation and depletion regimes of the semiconductor, respectively, which means that the depleted semiconductor layer prevents the formation of a stable polarization state^5^. This was modeled later on by Brondijk *et al.* for a FeFET device^16^. On the other hand, Kam *et al.* showed a close-to-full polarization reversal in MFS diodes using a small molecular semiconductor pentacene (30 nm layer thickness), and proposed the existence of a stable polarization state when the semiconductor is depleted, but only below the source-drain electrode regions in a bottom-gate top-contact FeFET^10^. Most recently, Gelinck *et al.* reported a close-to-full polarization reversal in a dual-gate FeFET using 20 nm inorganic indium-gallium-zinc oxide as the semiconductor, but only under certain biasing conditions, i.e., by applying a second gate bias to introduce a second charge accumulation layer. These authors state that this second accumulation layer causes the redistribution of electric field lines, which can effectively decrease the depolarization field and leads to a stable polarization state^15^.

Since several polarization states are proposed in FeFET devices when the semiconductors are depleted, namely depolarized or partially polarized, it is difficult to have a complete understanding of the general rules which control polarization and depolarization in FeFETs. A complicating factor is that the polarization might depend on the semiconductor layer thickness: indeed, analysis of the literature suggests that, the thinner the used layer, the more probable it is to obtain a stable polarization state in the depleted regime, although different semiconductors are employed. A possible reason would be that thinner semiconductor layers screen less the charges of the source and drain electrodes, which can thus partially compensate the polarization charges of the ferroelectric material. Another possibility is that, since the depleted semiconductor acts like an insulator which shares the poling voltage with the ferroelectrics, larger poling voltages, compared to the one applied to obtain the stable polarization when the semiconductor is in accumulation, are needed to reach the coercive field of P(VDF-TrFE). In addition, the location of P(VDF-TrFE), either over the source or drain regions, or over the channel region (Fig. 1c), may also affect the polarization behavior. These hypotheses do not obey the common belief that, in order to obtain a stable polarization of the ferroelectric material, a poling field sufficiently large compared to the coercive field is needed, together with polarization-compensating mobile charges in direct contact with the ferroelectric layer^17^.

These uncertainties largely result from the fact that there is currently no direct imaging of the switching of the transistor from its stable polarization state to its depolarized state, and more specifically of how the poling voltage distributes over the channel and electrode regions: although PFM has been used before to study the polarization and depolarization of P(VDF-TrFE) in capacitor-like structures^18^, direct application of PFM in a transistor architecture was not yet reported. In order to progress in theses issues, we therefore study for the first time by piezoresponse force microscopy (PFM) the local polarization and/or depolarization behavior in metal-semiconductor-ferroelectric (MSF) devices and unipolar FeFETs, for different semiconductor thicknesses and poling conditions. Then we fabricate and characterize unipolar FeFET devices, and check the influence of the semiconductor thickness and of the poling conditions on the device performance. Our microscopic studies reveal the complexity of the polarization state in the FeFET structure when the semiconductor is depleted, from which the behavior of FeFET devices can be fully explained and therefore improved.

## Results and Discussion

### PFM studies of Metal-Semiconductor-Ferroelectric (MSF) devices

**Figure 1:**
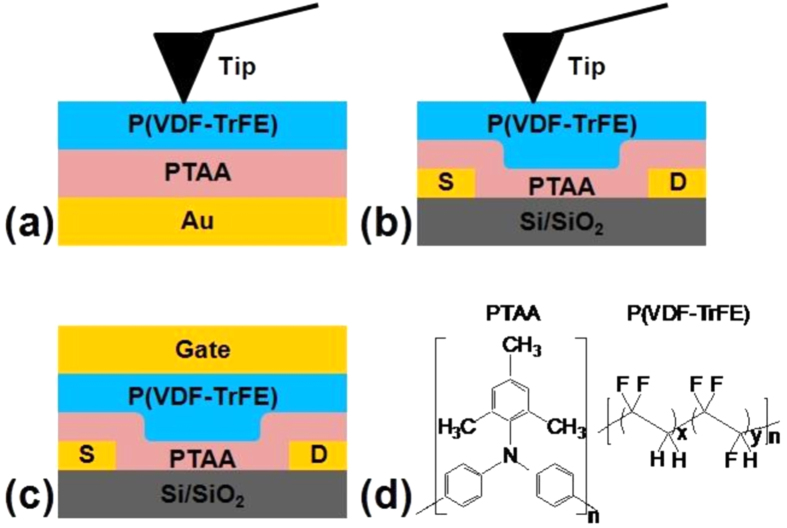
Schematic drawing of the device configurations used in this work. (**a**) A MSF sample using a mobile conductive tip as top electrode; (**b**) A top-gate bottom-contact FeFET using a mobile conductive tip as gate electrode; (**c**) A top-gate bottom-contact FeFET device used for current characterization. (**d**) Chemical structures of the organic materials.

**Figure 2:**
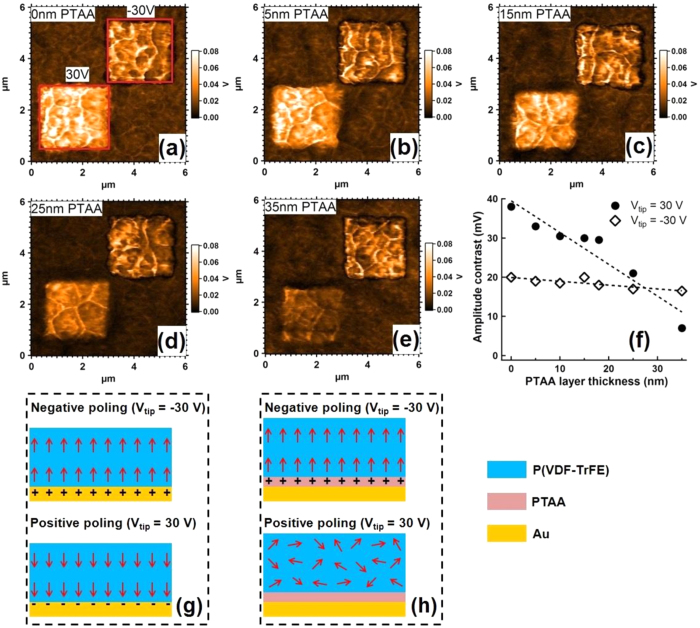
PFM amplitude images of MSF samples with a PTAA (**S**) layer thickness of (**a**) 0 nm, (**b**) 5 nm, (**c**) 15 nm, (**d**) 25 nm, and (**e**) 35 nm. On these samples, one small region (2 × 2 μm^2^, left-bottom) was poled by applying a 30 V tip bias and the other one (2 × 2 μm^2^, right top) a −30 V tip bias, while keeping the bottom electrode grounded. These two regions are boxed in red in (**a**). The PFM images were subsequently obtained at zero poling voltage over larger areas (6 × 6 μm^2^). The PFM amplitude contrasts between these two small regions and the unpoled background are shown in (**e**). Dashed lines are guides to the eye. Schematic descriptions of the polarization state after removal of the poling voltage are shown in (**g**) and (**h**), for zero and 35 nm PTAA layer thickness.

The polarization behavior of a P(VDF-TrFE) film of 200 nm thickness was first checked by PFM in MSF samples with a semiconducting PTAA layer of variable thickness (Fig. 1a). Fig. 2 shows PFM amplitude images corresponding to samples with PTAA layer thicknesses of 0, 5, 15, 25 and 35 nm. In all images, two small regions (2 × 2 μm^2^) were first poled by applying 30 V (left-bottom) or −30 V (right-top) to the conductive tip, while keeping the bottom electrode grounded. The PFM amplitude images were then obtained by scanning the tip on a larger area (6 × 6 μm^2^) while applying only an ac bias to the bottom electrode (1.5 V peak-to-peak amplitude) while keeping the tip grounded. The contrasts of PFM amplitude between the positively- or negatively-poled regions and the unpoled background are plotted in Fig. 2f as a function of PTAA layer thickness. The PFM amplitude contrast between poled and unpoled regions indicates that P(VDF-TrFE) is well-polarized and that the poled states are stable since the images are taken at zero poling bias well after poling. In these PFM amplitude images, a larger contrast indicates that more dipoles are aligned, with as a consequence a larger remnant polarization.

When the bias applied to the tip (*V*_*tip*_) is −30 V, the contrast is almost constant from 0 to 35 nm PTAA layer thickness, showing that the polarization behavior is hardly affected by the semiconducting layer (which is in accumulation state in this case); this is because the accumulation charges (holes) have been trapped at the interface and stabilize the ferroelectric polarization (Fig. 2h). However, the polarization behavior is completely different when *V*_*tip*_ is 30 V, in which case the PTAA layer is depleted. The contrast then decreases with increasing PTAA layer thickness and vanishes for 35 nm PTAA layer thickness. This observation is in good agreement with a previous result showing that there is no contrast for 45 nm PTAA layer thickness^19^. This decrease may *a priori* result from two different factors. First, the depleted PTAA layer acts like an insulator, which shares the poling bias with the P(VDF-TrFE) film; therefore, for a sufficiently thick PTAA layer, the electric field in the P(VDF-TrFE) layer might be too low to orient the dipoles during poling. Second, the PTAA layer increasingly screens the free electrons in the bottom metallic electrode, preventing them from fully compensating the polarization charges, resulting in depolarization after poling (Fig. 2h).

It is possible to decide which effect is dominant in our case; indeed, the voltage over the ferroelectric layer is *V*_F_* *= *VC*_S_/(*C*_S_ + *C*_F_), where *V* is the poling voltage, and *C*_F_ (resp. *C*_s_) is the capacitance of the ferroelectric (resp. semiconducting) layer. Taking ε_F_* *= 10 for the relative permittivity of the ferroelectric material and ε_S_* *= 3 for the one of the depleted PTAA layer^20^,^21^, the voltage over the 200 nm-thick ferroelectric layer when poling at 30 V decreases from 30 to 17.1 V when the PTAA layer thickness is increased from zero to 45 nm. This corresponds to an electric field from 150 to 64 MV/m, which is in all cases above the coercive field of our P(VDF-TrFE) layer (61.5 MV/m, see below). Therefore, the loss of polarization observed for the higher PTAA thickness does not result from incomplete poling of the ferroelectric, but from depolarization after poling due to the improper screening of the polarization charges by the too-distant free electrons of the metal. Nevertheless, a considerable polarization is still obtained when the PTAA layer is below *ca.* 20 nm, showing that two stable polarization states can be obtained in functional electronic devices if the semiconductor layer is thin enough not to screen fully the compensating charges of the metallic electrode.

### PFM studies of FeFET devices

In FeFET devices, the PTAA and P(VDF-TrFE) stacked layers are not always sandwiched between two metallic electrodes (Fig. 1c). Indeed, in the channel regions, the semiconducting/ferroelectric stack lies over an insulating region (SiO_2_ in our case), which is significantly different from a MFS device. Therefore, we further checked by PFM the local polarization switching in a FeFET device, using the configuration shown in Fig. 1b, again with different PTAA layer thicknesses and poling polarities.

**Figure 3:**
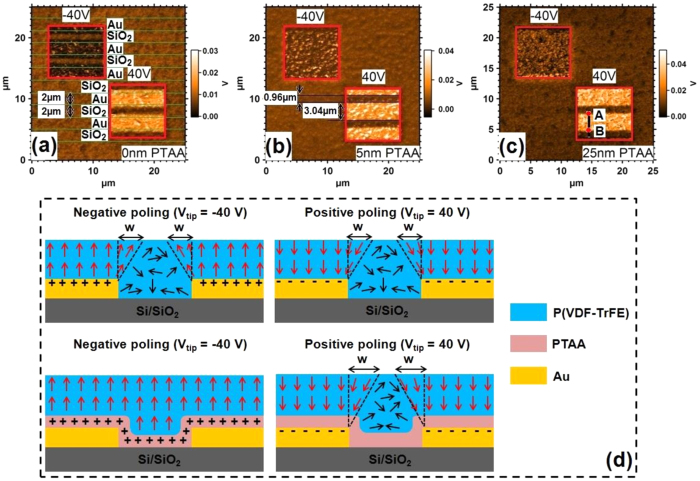
(**a–c**) PFM amplitude images of a 340 nm P(VDF-TrFE) film in a FeFET configuration with PTAA layers of different thickness. All images have two small regions (8 × 8 μm^2^, boxed in red) poled by applying −40 V or 40 V to the tip while keeping the source-drain electrodes grounded. Then the PFM images were obtained over a large region (25 × 25 μm^2^). (**a**) A reference sample with 0 nm PTAA layer. The underlying source-drain Au regions and SiO_2_ regions are indicated by green lines. The width of these two regions is 2 μm. (**b**) A sample with 5 nm PTAA layer. For 40 V tip bias, the width of the regions with strong and minor contrast with the background are 3.04 μm and 0.96 μm, respectively. (**c**) Same, for a 25 nm thick PTAA layer. Points A and B indicate the borders of the poled region with strong contrast. A cross section along the black line AB is drawn in the Supplementary Information for detailed analysis. (**d**) Schematic cross-section of a FeFET, showing the polarization after poling, depending on poling voltage polarity, in the absence or presence of a thin PTAA layer. Red arrows indicate the dipole moments in poled regions and black arrows are for unpoled regions. The compensating charges in the PTAA or electrodes are also indicated.

Fig. 3(a–c) shows the PFM amplitude images of FeFETs with 0 nm (as a reference), 5 nm, and 25 nm PTAA layer, respectively. 40 V poling biases of opposite polarity were applied over two small regions before imaging, as indicated; P(VDF-TrFE) is polarized when there is a PFM amplitude contrast between a poled region and the background – in this context, it is important to note that the contrast may be positive or negative, depending on the specific conditions on acquisition. Fig. 3(a–c) shows that the polarization depends on location. The source-drain Au electrode regions and the SiO_2_ channel regions, both of 2 μm width, are marked in Fig. 3a. Over the source and drain electrode regions, P(VDF-TrFE) is in a stable polarized state having a large contrast with the unpolarized background, irrespective of the polarity of the poling voltage and of whether a PTAA layer is or not present. This is consistent with the results obtained on MFS devices with thin PTAA layers.

However, the polarization behavior is different over the SiO_2_ channel regions. In the case of the reference FeFET without a PTAA layer (Fig. 3a), a significant portion of P(VDF-TrFE) is not polarized due to a dearth of compensation charges at the SiO_2_/P(VDF-TrFE) interface, whatever the polarity. When a PTAA layer is added in the device (Fig. 3(b–c)), the polarization over the channel regions now depends on the polarity of the poling voltage. For a negative bias (−40 V), the P(VDF-TrFE) can be polarized everywhere, since the PTAA layer is in accumulation, acting as an electrode during poling and providing the holes needed to stabilize the polarization after removal of the poling voltage. In contrast, when a positive bias (40 V) is applied, the central portion of the channel regions is not polarized, because the applied bias is not exceeding the coercive voltage of P(VDF-TrFE) in these regions.

Nevertheless, in the case of the positive poling voltage for all PTAA thicknesses, and for the negative poling voltage only for the reference device without PTAA, a narrow stripe of P(VDF-TrFE) resting over the insulating channel regions is also polarized over a width *w* in the vicinity of the source-drain electrode regions. This can be seen from the width of the polarized and unpolarized regions, compared to the width of the source-drain electrodes and of the channel (both equal to *w*_e_* *= 2 μm): for instance, in Fig. 3b, the width of the poled regions is 3.04 μm while the width of the unpoled ones is 0.96 μm; hence, there is a stripe of width *w *= (3.04–2)/2 = 0.52 μm of poled P(VDF-TrFE) lying over both edges of the channel region.

**Figure 4:**
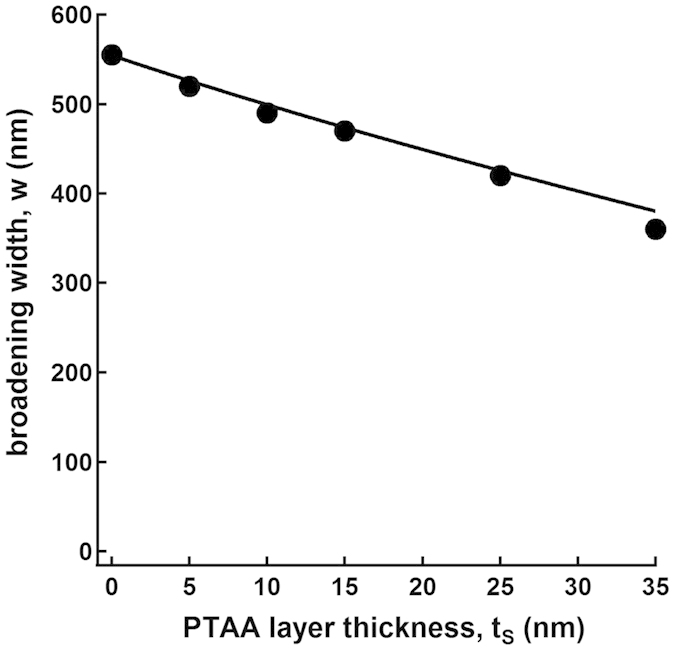
Width of the poled stripe at the edge of the electrodes over the channel regions, *w*, versus PTAA layer thickness, *t*_S_, for the FeFETs of Figure 3 comprising a ferroelectric layer of 340 nm thickness, after positive poling at 40 V. The closed circles are the experimental data; the continuous line is the prediction from the model (equation 1).

The permanently-polarized regions are defined by the regions over which the electric field in the ferroelectric layer during poling is larger than the coercive field; they are shown schematically in Fig. 3d for the different cases considered here. A detailed analysis of this phenomenon needs to take into account the division of the poling voltage between the insulating depleted PTAA layer and the ferroelectric layer, and allows to obtain the size and shape of the polarized regions. From this analysis (in the Supplementary Information), the coercive field *E*_c_ of the P(VDF-TrFE) layer is found to be 61.5 MV/m, and the broadening of the polarized regions at the edge of the electrodes, *w*, is computed to be:


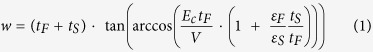


with *t*_F_ and *t*_S_ the thickness of the ferroelectric and semiconducting layers, respectively, *V* the applied poling voltage, and ε_S_ (3) and ε_F_ (10) the relative permittivities of the semiconducting and ferroelectric materials, respectively. This equation represents properly the broadening of the poled regions seen by PFM at the top surface of the ferroelectric layer (Fig. 4).

**Figure 5:**
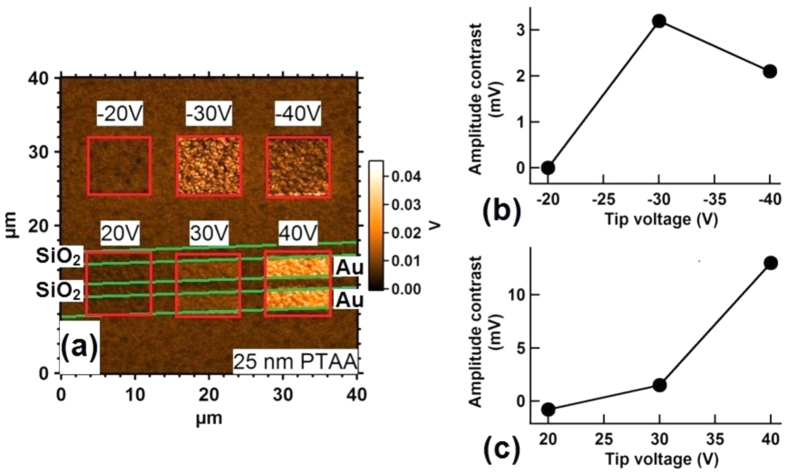
PFM response of a P(VDF-TrFE) film in a FeFET configuration with a 25 nm-thick PTAA layer, measured for different poling voltages. (**a**) PFM amplitude image with six small regions poled by applying different tip voltages (8 × 8 μm^2^). The poled regions are boxed in red and the applied tip voltages are marked on top of each red box. The underlying Au and SiO_2_ regions are the same as in Fig. 3, and are therefore not marked on the image. The PFM amplitude contrast between the poled regions and the unpoled background region is plotted in panels (**b,c**).

During the poling at positive voltages, the depleted PTAA layer thus acts as an insulator and voltage divider; therefore, the effective voltage on the P(VDF-TrFE) is lower than the total applied poling voltage, and the poled regions decrease in size for thicker PTAA layers, as Fig. 4 clearly shows. This effect defines the shape and size of the poled regions. However, after positive poling, the depleted PTAA layer plays a second role, which is to screen the compensating charges provided by the metallic electrodes, as was seen in the MSF devices. Due to incomplete compensation, the poled regions partially depolarize, resulting in a lower PFM contrast. This was confirmed by checking the polarization of P(VDF-TrFE) after application of different poling voltages to a FeFET comprising a 25 nm-thick PTAA layer (Fig. 5). When the PTAA layer is in accumulation mode and plays the role of an electrode, the PFM contrast between poled and unpoled regions increases for more negative poling voltages, reaching saturation below −30 V poling voltage (the ‘coercive voltage’ is about −21 V for a 340 nm-thick ferroelectric layer). However, when the PTAA layer is depleted, the PFM contrast in the poled regions is far from saturation at 30 V, which is essentially due to depolarization after poling since our previous model shows that the coercive field is attained above the electrodes in these conditions.

At this stage, we have thus developed a fine understanding of polarization in FeFETs during and after poling. When the semiconducting PTAA is in accumulation, a stable upward polarization state is obtained over the complete device during and after poling, because the PTAA layer acts as an electrode during poling, and subsequently provides free holes which compensate the polarization charges at the P(VDF-TrFE)/PTAA interface. In this case, the thickness of PTAA layer has minor influence on the polarization behavior and the saturation polarization is obtained for the same poling voltage, whatever the PTAA thickness (30 V is enough for 340 nm P(VDF-TrFE) with our processing conditions). When the semiconducting PTAA is depleted, the poling efficiency is reduced, because the voltage is divided between the insulating PTAA layer and the ferroelectric layer; therefore, poled regions are limited to the regions shown in Fig. 3d and described by equation 1. However, even though the internal poling field is high enough in these regions, their polarization is partially lost when the poling field is removed, because the compensating charges provided by the metallic electrodes are not in direct contact with the ferroelectric layer, which corresponds to increased electrostatic energy as shown by the study of the MSF systems. Therefore, thinner PTAA layers (typically below *ca.* 20 to 25 nm) are needed if two stable polarization states of opposite polarity are desired. In addition, we also checked these two polarization states right after and one day after the poling; no significant decreasing of the amplitude contrast was observed, indicating these two polarization states are stable over long times (Figure S2 in the Supplementary Information).

### Characterization of complete FeFET devices

**Figure 6:**
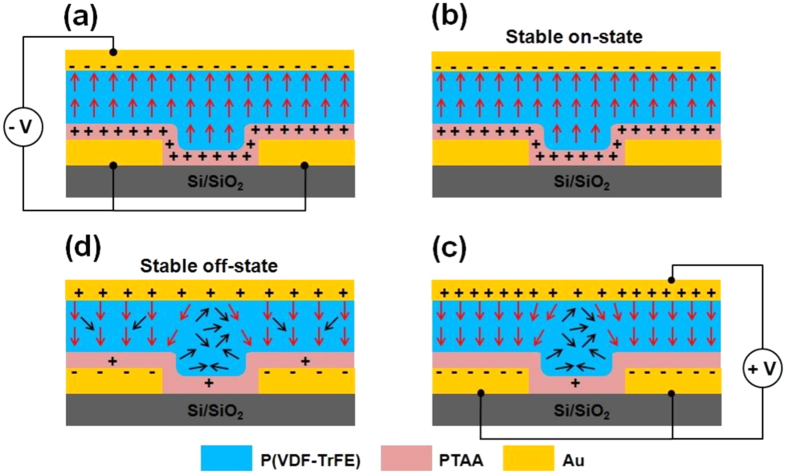
Schematic drawing of the state of a FeFET at different stages of its poling. The red arrows indicate poled regions, black arrows showing the local dipole moments in depolarized regions. Compensating charges are also indicated.

The previous experimental results can now be used to describe the behavior of complete FeFET devices (Fig. 1c). When the FeFET is poled negatively, the ferroelectric layer is completely upward-polarized provided the poling voltage corresponds to an electric field larger than *E*_c_ (Fig. 6a). When the poling voltage is decreased to zero, the polarization is essentially conserved, corresponding to the stable “on”-state with polarization-compensating holes accumulated in the semiconducting layer and therefore a high channel conductance (Fig. 6b). When a positive poling voltage is applied onto this stable state, the polarization state of the ferroelectric switches downwards at a given switching voltage *V*_dp_, over the metallic electrodes and in a narrow edge region over the channel described by equation 1. The extent of polarization depends on the conditions of poling. In the rest of the channel region, the ferroelectric depolarizes due to the lack of compensating charges in the now-depleted semiconducting channel (Fig. 6c). This corresponds to a strongly-depleted semiconducting channel, and a low current. When the poling voltage is subsequently decreased to zero, the ferroelectric polarization in the poled region partially depolarizes depending on the thickness of the PTAA layer. Hence, the stable “off”-state is one into which, depending on the thickness of the PTAA layer, a partially-polarized region exists over the electrodes and close to them, whereas the material is unpolarized in the remaining part of the device over the channel (Fig. 6d). Depending on the extent of depolarization and thus on the PTAA thickness, the semiconducting channel will be more or less depleted, with a more or less low channel conductance. This description is valid for a p-type semiconducting layer such as PTAA; it should be reversed if a n-type semiconductor is used.

**Figure 7:**
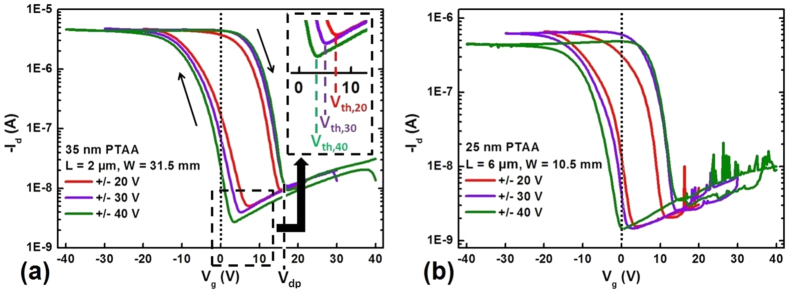
Transfer curves of two FeFETs with different source-drain configuration and PTAA layer thickness. Both graphs present three transfer curves with different gate sweeping voltage ranges of ± 20 V, ± 30 V and ± 40 V, by applying a same source-drain voltage *V*_*d*_ of −5 V. A dotted vertical line at 0 V is drawn as reference in both graphs. (**a**) FeFET with a 35 nm-thick PTAA layer and a source-drain configuration with *L *= 2 μm and *W *= 31.5 mm. The threshold voltages *V*_*th*_ in the case of different sweeping voltage ranges are indicated in the enlarged insertion panel. Black arrows indicate the gate voltage sweeping direction. The depolarization voltage (*V*_*dp*_) from the upwards saturated polarization state corresponding to the accumulation regime of PTAA is also indicated. (**b**) FeFET with a 25 nm-thick PTAA layer and a source-drain configuration with *L *= 6 μm and *W *= 10.5 mm.

FeFET devices were then fabricated with a P(VDF-TrFE) layer of 340 nm, while the semiconducting PTAA layer thickness was different for each device, ranging from 10 nm to 35 nm. Fig. 7a shows three series of transfer curves obtained by applying different poling gate voltages of a FeFET with a channel length *L *= 2 μm, a channel width *W *= 31.5 mm and a 35 nm semiconducting PTAA layer. All curves exhibit hysteresis loops which are typical for memory FeFETs.

Starting from the negatively polarized state, the channel current is preserved at a high value when the gate voltage is increased to the stable on-state at 0 V, corresponding to moving from (a) to (b) in Fig. 6. When increasing further the gate voltage to positive values, the current decreases abruptly at *V*_dp_, at which stage the ferroelectric depolarizes in the channel region (Fig. 6c), resulting in a low channel current. Depolarization occurs for a lower value of voltage when the transistor was initially poled at −20 V (red curve), because the saturation of the polarization was not reached for this poling voltage; however, saturation is obtained when poling at −30 and −40 V (Fig. 5b), with as a result an identical depolarization voltage (green and purple curves).

When sweeping back the gate voltage to zero, the current increases again below a threshold voltage *V*_th_, which is due to partial depolarization of the ferroelectric over and close to the electrodes (Fig. 6d). Interestingly, as shown in the inset of Fig. 7a, the threshold voltage is lower when using higher poling voltages, which results from three effects: first, higher poling voltages correspond to larger poled regions in the depletion regime (Fig. 5); second, higher poling voltages also correspond to more polarized poled regions (Fig. 5c) – at least within the 30–40 V poling voltage range; third, this more polarized state makes PTAA more depleted and increases the charge injection barrier between the source-drain electrode and PTAA (a simplified band diagram of the Au/PTAA/P(VDF-TrFE) structure is shown in Figure S3 of the Supplementary Information)^22^,^23^.

Less positive *V*_*th*_’s are beneficial, because they increase the on/off current ratio at zero gate bias which is an important operational parameter for FeFET memory devices. For instance, the on/off ratio of the FeFET of Fig. 7a is increased by a factor of 10 by changing the positive poling voltage from 20 V to 40 V.

**Figure 8:**
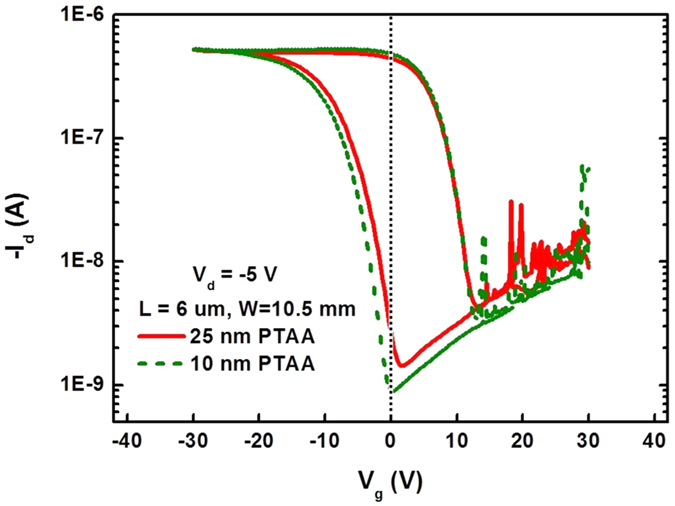
Transfer curves of two FeFETs with a 10 and 25 nm PTAA layer, respectively. A dotted vertical line at 0 V is drawn as reference.

Fig. 7b shows transfer curves of a FeFET employing a different device configuration (*L *= 6 μm, *W *= 10.5 mm) and a PTAA layer of different thickness (25 nm) compared to that in Fig. 7a. A generally similar behavior as in Fig. 7a is observed, indicating that our analysis is independent of the FeFET configuration and of the semiconducting PTAA layer thickness. The current in the on-state is lower due to the lower channel conductance. Importantly, the threshold voltage in Fig. 7b is less positive (and even negative) than in Fig. 7a; this results from the thinner PTAA layer, leading to a wider poled region of lower degree of depolarization, resulting in a more depleted PTAA layer and a larger charger injection barrier. The importance of the PTAA thickness was also checked by using the same device configuration: Fig. 8 presents the transfer curves of two FeFETs with *L *= 6 μm and *W *= 10.5 mm, but of different PTAA layer thickness (10 and 25 nm). The poling voltage sweeping range of the two FeFETs was the same (±30 V). As expected, *V*_*th*_ is less positive for the device with a 10 nm PTAA layer, because the poled regions are larger and more polarized. However, the right part of the curves coincides, indicating identical depolarization voltages, because the same saturated polarization on-state is obtained by applying a −30 V gate poling voltage.

The present results demonstrate that thinner semiconducting layers give larger downwards polarizations and therefore better device performance. However, the semiconducting layer thickness might also affect the film quality and therefore the semiconducting properties. Figure S4 in the Supplementary Information shows that a flat surface with a roughness smaller than 0.2 nm is obtained for a 10 nm PTAA layer. In contrast, PTAA films less than 10 nm exhibit a large amount of defects, probably due to the fast evaporation of the solvent during the spin-coating. Therefore, the optimized PTAA layer thickness should be in the range of 10 to 20 nm in order to obtain a considerable downwards polarization state and a large on/off ratio at zero gate bias. A further factor to take into account is that the mobility of the semiconductors (especially small molecular ones) strongly depends on the film thickness^24^,^25^.

We thus conclude that, when the PTAA layer is depleted, the spatial extent and magnitude of the polarization over the source-drain electrodes and neighboring regions modulate the position of the threshold voltage, resulting in higher or lower on/off current values at zero gate bias. The on/off ratio can be increased by up to 2 orders of magnitude in our case by carefully choosing the PTAA layer thickness, e.g., 10 nm compared to 45 nm. Similar degrees of improvement of the on/off ratio were also reported recently^21^; in that work, a 80 nm PTAA layer was used and a second gate was employed to control the threshold voltage and the on/off current ratio. Our work shows that tuning the PTAA layer thickness affords another handle to control the on/off ratio, with the advantage of being intrinsic and not requiring to use a second controlling gate.

## Conclusions

In summary, we have systematically studied the local ferroelectric polarization and/or depolarization of P(VDF-TrFE) in MFS and unipolar top-gate bottom-contact FeFET devices using p-type poly(triarylamine) as organic semiconductor. PFM results show that a stable “on” upward-pointing polarization state can be obtained irrespective of the location in FeFETs of P(VDF-TrFE) (on channel regions or source-drain electrode regions), in which the semiconducting layer acts as an electrode during poling and provide compensating charges at the PTAA/P(VDF-TrFE) interface after removal of the poling voltage. Another stable state is also achievable in these devices when the depleted PTAA layer is thin enough and/or the poling voltages are sufficiently large. In this state, the ferroelectric is depolarized over the main part of the channel regions, whereas it is polarized downwards over the electrodes and neighboring channel regions. The size of the polarized regions depends on the thickness of the semiconducting layer, because PTAA acts as an insulator dividing the applied voltage during poling; in addition, their degree of polarization after removal of the poling voltage also depend on the thickness of the PTAA layer, which screens the polarization-compensating charges accumulated in the source and drain electrodes. These polarized regions in the second stable state make the PTAA more depleted and modify the charge injection barrier between the electrode and the PTAA layer. As a result, FeFET devices of thinner PTAA layer have less positive (and even negative) threshold voltages, leading to an increase of the on/off current ratio at zero gate bias and therefore to an improvement of the memory device performance.

The semiconductor thickness dependence of the ferroelectric polarization in a FeFET is likely to be universal, because similar polarization behaviors were reported for other organic/inorganic semiconductors^10^,^15^. Therefore, our investigation and results provide new clues for device design and performance optimization of memory FeFETs. In addition, our results give a complete spatial view – and thereby understanding of - ferroelectric polarization and depolarization in these complex devices, and on the working mechanism of memory FeFETs, as summarized in Fig. 6.

## Methods

### Materials

P(VDF-TrFE) (65 wt% of VDF units and a weight-average molar mass of *ca.* 300 000 g/mol, Fig. 1d) was provided by Solvay Specialty Polymers. Poly(triarylamine) (PTAA, a p-type amorphous organic semiconductor, Fig. 1(d)) was purchased from Flexink. P(VDF-TrFE) was dissolved in acetylacetone (65 g/L). PTAA was dissolved in toluene (2, 5, and 10 g/L). All solutions were filtered through PTFE filters with 0.45 μm pore size before spin-coating.

### Device Fabrication

MSF devices (Fig. 1 (a)) were fabricated on a Si/SiO_2_ substrate covered by a 10/100 nm thick Cr/Au layer. PTAA layers of 5–45 nm thickness were spin-coated from solutions of different concentration using various spin speeds. On top of the PTAA layers, *ca.* 200 nm of P(VDF-TrFE) was spin-coated (5000 rpm, 500 r/s, 1 min) from a 65 g/L P(VDF-TrFE) solution. We note that acetylacetone, used to dissolve P(VDF-TrFE), is an orthogonal solvent to the underlying PTAA layer. The resulting samples were annealed at 135 °C for 10 min in air to improve the crystallinity of P(VDF-TrFE). A conductive tip was used as the top electrode when characterizing the devices in a PFM setup.

For FeFET devices (Fig. 1 (b) and (c)), 5/30 nm thick Cr/Au source-drain electrodes were defined by standard photolithography on a Si (100) substrate covered by 200 nm of thermal SiO_2_. On this substrate, 10–45 nm thick PTAA layers were spin-coated from PTAA solutions of different concentration using various spin speeds. On top of the PTAA layers, *ca.* 340 nm of P(VDF-TrFE) was spin-coated (2000 rpm, 500 r/s, 1 min) from a 65 g/L P(VDF-TrFE) solution. The resulting samples were annealed at 135 °C for 10 min in air. In the case of devices tested in PFM, a conductive tip was used as top electrode; in the case of devices for current characterization, 70 nm-thick Au top electrodes were evaporated through a shadow mask.

### Device Characterization

The piezoresponse of the P(VDF-TrFE) films in MSF and FeFET devices was checked by PFM (Bruker Icon Dimension) in contact mode. The PFM technique allows to pole P(VDF-TrFE) and check the polarization on any desired position. To pole the device, a dc bias was applied to the conductive tip during scanning, while keeping the bottom electrodes grounded. An ac bias (1.5 V peak-to-peak amplitude) was then applied to the bottom electrodes to image the piezo/ferroelectric response, while keeping the tip grounded. The conductive Si tip was purchased from Nanosensors (Boron-doped diamond-coated, force constant of 0.02−0.77 N/m).

The current characterization of the FeFET devices was performed in air by using an Agilent B1500A semiconductor device analyzer equipped with a probe station. To obtain the transfer curves of FeFETs, a −5 V source-drain voltage was applied while sweeping the gate voltage from a positive value to a negative value, and to a positive value again. The devices were operated in the linear regime with these operation conditions^19^.

## Additional Information

**How to cite this article**: Cai, R. and Jonas, A. M. Local Maps of the Polarization and Depolarization in Organic Ferroelectric Field-Effect Transistors. *Sci. Rep.* **6**, 22116; doi: 10.1038/srep22116 (2016).

## Supplementary Material

Supplementary Information
