# Supplementary material for: Local Maps of the Polarization and Depolarization in Organic Ferroelectric Field-Effect Transistors
Source: Sci Rep. 2016 Feb 24;6:22116. doi: 10.1038/srep22116 (PMC4764979; doi:10.1038/srep22116)
Supplement: Supplementary Information [file srep22116-s1.pdf]

Local Maps of the Polarization and Depolarization in Organic Ferroelectric Field-Effect Transistors

Ronggang Cai* and Alain M. Jonas*

Bio & Soft Matter, Institute of Condensed Matter and Nanosciences, Université catholique de Louvain, Croix du Sud 1/L7.04.02, B1348 Louvain-la-Neuve, Belgium

*Corresponding authors:

Ronggang Cai (ronggang.cai@uclouvain.be)

Alain M. Jonas (alain.jonas@uclouvain.be)

1. Computation of the layer thicknesses and fields in the poling region in FeFET devices.

A cross-section of a FeFET device is shown in Figure S1.

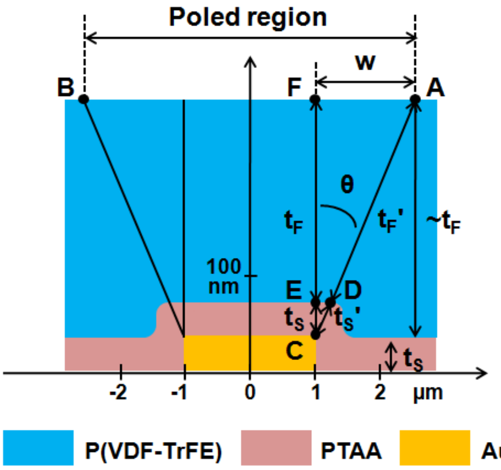

Figure S1. Schematic drawing of the cross-section of a FeFET device. The AC line indicates the boundary of the region beyond which the electric field during poling is below the coercive field.

Previous AFM experiments performed for thin PTAA layers (up to 45 nm thick) spin-coated over protruding gold electrodes showed that the PTAA layer conformally covers the electrodes;[1] what is not known, however, is the exact profile of the PTAA layer at the edge of the electrodes. As for the P(VDF-TrFE) layer, it is thick enough to planarize the structure. Points A and B in Figure S1 are the positions where the PFM contrast starts to appear, *i.e.*, at which the coercive field is reached when a poling voltage of 40 V is applied (Figure 3 (c) of the article). Stated otherwise, the electric field in P(VDF-TrFE) is equal to the coercive field along the line AC of Figure S1. Based on the geometry of the cross-section, on the ferroelectric and semiconducting layer thicknesses (t_F=340 nm and t_S = 0 to 35 nm, respectively), and on the measured value of broadening of the poled region (w, Table S1), it is possible to compute the voltage drop *in the ferroelectric layer* along the AC line, $V_{AD} = t_F' E_c$, where E_c is the coercive field and t_F' is the thickness of the ferroelectric layer along the AC line (Figure S1).

In the absence of a PTAA layer (Figure 3 (a) of the article), the voltage drop V_{AD} is the applied voltage (40 V); since w = 555 nm and t_S = 0, t_F' = 651 nm, which provides

an experimental value for the coercive field E_c = 61.5 MV/m, close to the reported values of coercive field for relatively thick P(VDF-TrFE) films.[2, 3]

In the presence of a PTAA layer thin enough not to fully screen the compensating charges from the electrodes, the following two equations may be solved to obtain t_F' and t_S', which are the thicknesses of the ferroelectric and semiconducting layers along the AC line (Figure S1):

$$t_F' + t_S' = \sqrt{(t_S + t_F)^2 + w^2}$$

$$V_{AD} = E_c t_F' = V \frac{\epsilon_S / t_S'}{(\epsilon_S / t_S') + (\epsilon_F / t_F')},$$

where V is the applied poling voltage (40 V), and ε_S = 3 (resp. ε_F = 10) is the relative permittivity of the semiconducting (resp. ferroelectric) layer. The first equation is purely geometrical, whereas the second expresses that the applied poling voltage is shared between two series capacitors and that the electric field on the ferroelectric layer at the limit of the polarization zone is equal to the coercive field.

The resulting thickness values are collected in Table S1, from which the voltage drops $V_{AD} = t_F' E_c$ and $V_{DC} = V - V_{AD}$ can be trivially computed. These voltage drops are very close to the values of voltage drops in flat MSF devices of same layer thickness, which indicates that the voltage division is essentially not perturbed by edge effects in the poled region. Therefore, it becomes possible to simplify the estimation of the width of the poled region, w. Defining θ as the angle between the AC line and the vertical direction (Figure S1),

$$w = (t_S + t_F)\tan(\theta)$$

$$E_c = \frac{V_{AD}}{t_F'} \approx \frac{V_{EF}}{(t_F / \cos\theta)},$$

in which $V_{EF} = \frac{V}{1 + (\epsilon_F / \epsilon_S) \cdot (t_S / t_F)}$ is the voltage drop in the ferroelectric layer over the electrodes, far from their edges (as in a MSF device). Therefore,

$$w \approx (t_{\mathrm{F}} + t_{\mathrm{S}})\tan\left(\arccos\left(\frac{E_c t_{\mathrm{F}}}{V}\cdot(1+\frac{\epsilon_{\mathrm{F}}}{\epsilon_{\mathrm{S}}}\frac{t_{\mathrm{S}}}{t_{\mathrm{F}}})\right)\right)$$

$$= (t_{\mathrm{F}} + t_{\mathrm{S}})\sqrt{\frac{V^2}{E_c{}^2 t_{\mathrm{F}}{}^2\cdot(1+\frac{\epsilon_{\mathrm{F}}}{\epsilon_{\mathrm{S}}}\frac{t_{\mathrm{S}}}{t_{\mathrm{F}}})^2}-1} \quad \text{(equation S1).}$$

A comparison between the measured and computed values of *w* is given in Table S1 and Figure 4 of the companion article. The measured and computed values agree very well, within better than 20 nm, confirming the predictive ability of equation S1 when using E_c=61.5 MV/m, ε_S = 3 and ε_F = 10.

Table S1: Different geometrical and electrical parameters obtained from the analysis of PFM images of poled FeFET devices.

$t_S^{(1)}$ (nm)	$w^{(2)}$ (nm)	$t_F'^{(3)}$ (nm)	$t_S'^{(4)}$ (nm)	$V_{AD}^{(5)}$ (V)	$V_{DC}^{(6)}$ (V)	$w_{calc}^{(7)}$ (nm)
0	555	651	0	40	0	554
5	520	615	9	37.8	2.2	526
10	490	585	17	36.0	4.0	499
15	470	564	25	34.7	5.3	474
25	420	518	38	31.9	8.1	426
35	360	471	49	29.0	11.0	380

(1) Measured thickness of the PTAA layer; (2) Measured broadening of the P(VDF-TrFE)-polarized region on one side of the source or drain electrodes; (3) Computed distance DA in Figure S1; (4) Computed distance CD in Figure S1; (5) Computed voltage drop over the ferroelectric layer, along the line AC of Figure S1; (6) Computed voltage drop over the semiconductor layer, among the line AC of Figure S1; (7) Computed broadening of the P(VDF-TrFE) region, equation S1.

2. Stability of the two polarization states

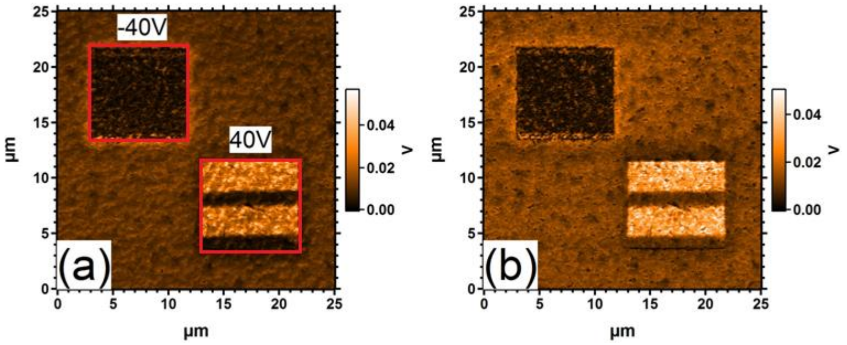

Figure S2: PFM amplitude images of a FeFET with 15 nm PTAA and 340 nm P(VDF-TrFE) which was poled on two small regions by -40 V (left-top) and 40 V (right-bottom) tip bias. (a) The image was taken right after the poling; (b) The image was taken one day after the poling and no significant decreasing of the amplitude contrast is observed compared to that in (a), showing that the two polarization states in our FeFET are stable over long times.

3. Simplified band diagram of the metal/semiconductor/ferroelectrics structure

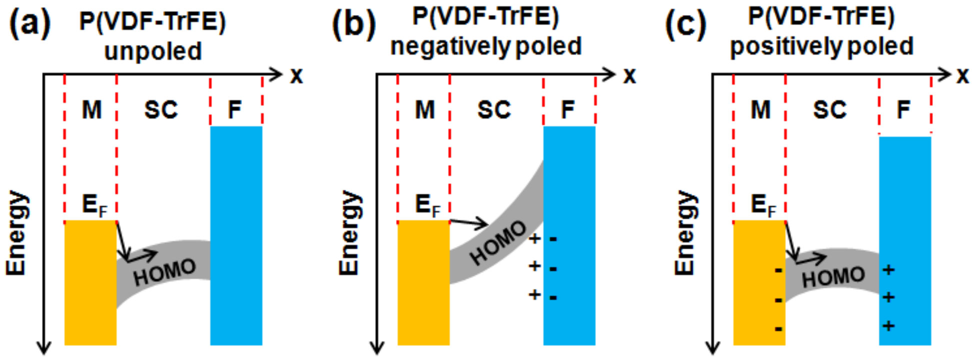

Figure S3. Band diagram of the Au/PTAA/P(VDF-TrFE) (M/SC/F)structure, taking 4.8 eV for the work function of Au,[4] and 5.2-5.6 eV for the HOMO level of PTAA.[5] (a) P(VDF-TrFE) is unpoled; (b) P(VDF-TrFE) is negatively poled and PTAA is in the accumulation state; (c) P(VDF-TrFE) is positively poled and PTAA is in the depletion state.

4. AFM images of thin PTAA layers

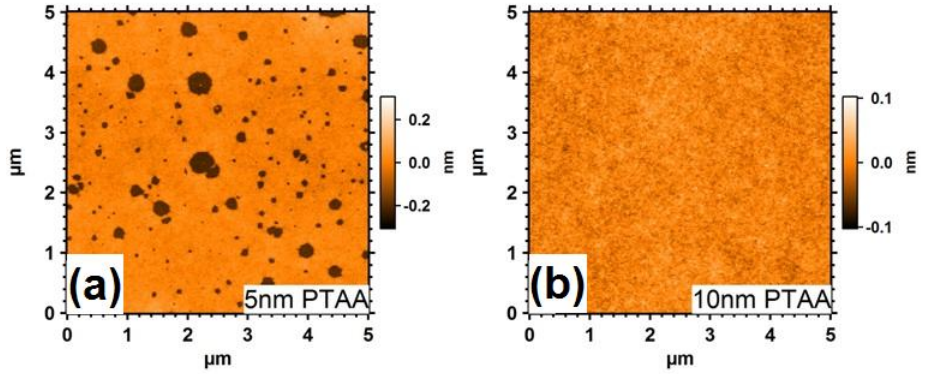

Figure S4: Topography images of two PTAA layers spin-coated on SiO_2. The PTAA layer thickness is (a) 5 nm and (b) 10 nm.

5. PFM characterization of pristine P(VDF-TrFE)

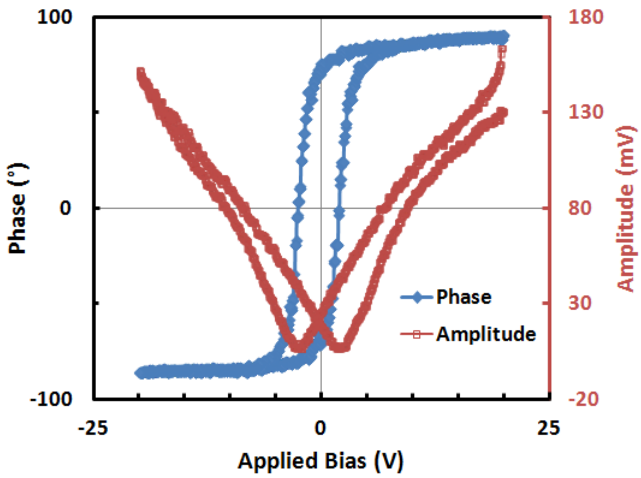

Figure S5: PFM phase and amplitude as a function of the applied bias on a 200 nm-thick P(VDF-TrFE) film using Au as bottom electrode and the PFM tip as top electrode. A square PFM phase hysteresis loop and butterfly-shaped amplitude curve are obtained.

References

1. Cai, R. *et al.* An organic ferroelectric field effect transistor with poly(vinylidene fluoride-co-trifluoroethylene) nanostripes as gate dielectric. *Appl. Phys. Lett.* **105,** 113113 (2014).

2. Naber, R. C. G. *et al.* Origin of the drain current bistability in polymer ferroelectric field-effect transistors. *Appl. Phys. Lett.* **90,** 113509 (2007).

3. Gelinck, G. H., van Breemen, A. J. J. M. & Cobb, B. Ferroelectric switching of poly(vinylidene difluoride-trifluoroethylene) in metal-ferroelectric-semiconductor non-volatile memories with an amorphous oxide semiconductor. *Appl. Phys. Lett.* **106,** 093503 (2015).

4. Asadi, K., de Boer, T. G., Blom, P. W. M. & de Leeuw, D. M. Tunable Injection Barrier in Organic Resistive Switches Based on Phase-Separated Ferroelectric–Semiconductor Blends. *Adv. Funct. Mater.* **19,** 3173-3178 (2009).

5. Logan, S., Donaghey, J. E., Zhang, W., McCulloch, I. & Campbell, A. J. Compatibility of amorphous triarylamine copolymers with solution-processed hole injecting metal oxide bottom contacts. *J. Mater. Chem. C* **3,** 4530-4536 (2015).
